# *O*-Ethyl *S*-{(*S*)-1-oxo-1-[(*R*)-2-oxo-4-phenyl­oxazolidin-3-yl]propan-2-yl} carbonodi­thio­ate

**DOI:** 10.1107/S1600536814007636

**Published:** 2014-04-18

**Authors:** J. Pablo García-Merinos, Heraclio López-Ruiz, Yliana López, Susana Rojas-Lima

**Affiliations:** a Área Académica de Química, Universidad Autónoma del Estado de Hidalgo, Carretera Pachuca-Tulancingo Km. 4.5, Mineral de La Reforma, Hidalgo, CP 42076, Mexico; b Instituto de Investigaciones Químico-Biológicas, Universidad Michoacana de San Nicolás de Hidalgo, Morelia, Michoacán, CP 58000, Mexico

## Abstract

In the title compound, C_15_H_17_NO_4_S_2_, synthesized by addition of *O*-ethylxanthic acid potassium salt to a diastereomeric mixture of (4*R*)-3-(2-chloro­propano­yl)-4-phenyl­oxazolidin-2-one, the oxazolidinone ring has a twist conformation on the C—C bond. The phenyl ring is inclined to the mean plane of the oxazolidinone ring by 76.4 (3)°. In the chain the methine H atom is involved in a C—H⋯S and a C—H⋯O intra­molecular inter­action. In the crystal, mol­ecules are linked by C—H⋯π inter­actions, forming chains along [001]. The *S* configuration at the C atom to which the xanthate group is attached was determined by comparison to the known *R* configuration of the C atom to which the phenyl group is attached.

## Related literature   

For the use of chiral oxazolidinones auxiliaries in asymmetric synthesis, see: Evans (1982 ▶); Ager *et al.* (1997 ▶). For the oral activity of oxazolidinonas against multidrug-resistant Gram-positive bacteria, see: Müller & Schimz (1999 ▶). For our work on the synthesis of novel heterocyclic compounds, see for example: López-Ruiz *et al.* (2011 ▶). For the crystal structures of similar compounds, see: Bartczak *et al.* (2001 ▶); Kruszynski *et al.* (2001 ▶); Wouters *et al.* (1997 ▶). For the crystal structures of 3,4-disubstituted oxazolidinone derivatives, see: Marsh *et al.* (1992 ▶); Evain *et al.* (2002 ▶); Hwang *et al.* (2006 ▶). For standard bond lengths, see: Allen *et al.* (1987 ▶). For ring puckering analysis, see: Cremer & Pople (1975 ▶).
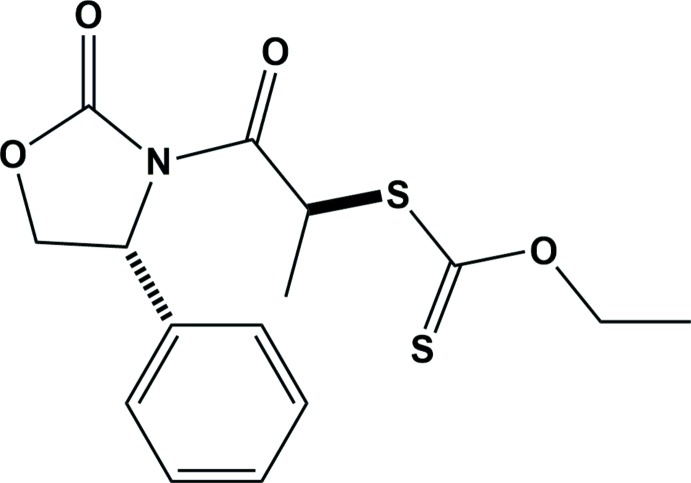


## Experimental   

### 

#### Crystal data  

C_15_H_17_NO_4_S_2_
*M* *_r_* = 339.42
Monoclinic, 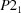

*a* = 10.8558 (15) Å
*b* = 6.1867 (9) Å
*c* = 12.3057 (17) Å
β = 94.911 (4)°
*V* = 823.4 (2) Å^3^
*Z* = 2
Mo *K*α radiation
μ = 0.34 mm^−1^
*T* = 293 K
0.2 × 0.17 × 0.16 mm

#### Data collection  

Bruker SMART CCD diffractometer
10191 measured reflections
3223 independent reflections
1681 reflections with *I* > 2σ(*I*)
*R*_int_ = 0.152

#### Refinement  

*R*[*F* ^2^ > 2σ(*F* ^2^)] = 0.067
*wR*(*F* ^2^) = 0.191
*S* = 0.87
3223 reflections
201 parameters
1 restraint
H-atom parameters constrained
Δρ_max_ = 0.38 e Å^−3^
Δρ_min_ = −0.35 e Å^−3^
Absolute structure: Flack (1983 ▶), 6968 Friedel pairs
Absolute structure parameter: 0.08 (18)

### 

Data collection: *SMART* (Bruker, 1999 ▶); cell refinement: *SAINT* (Bruker, 1999 ▶); data reduction: *SAINT*; program(s) used to solve structure: *SHELXS97* (Sheldrick 2008 ▶); program(s) used to refine structure: *SHELXL97* (Sheldrick, 2008 ▶); molecular graphics: *PLATON* (Spek, 2009 ▶) and *Mercury* (Macrae *et al.*, 2008 ▶); software used to prepare material for publication: *WinGX* (Farrugia, 2012 ▶) and *publCIF* (Westrip, 2010 ▶).

## Supplementary Material

Crystal structure: contains datablock(s) I. DOI: 10.1107/S1600536814007636/su2715sup1.cif

Structure factors: contains datablock(s) I. DOI: 10.1107/S1600536814007636/su2715Isup2.hkl

Supporting information file. DOI: 10.1107/S1600536814007636/su2715Isup3.cml

CCDC reference: 995594

Additional supporting information:  crystallographic information; 3D view; checkCIF report

## Figures and Tables

**Table 1:** Hydrogen-bond geometry (Å, °) *Cg* is the centroid of the C10–C15 phenyl ring.

*D*—H⋯*A*	*D*—H	H⋯*A*	*D*⋯*A*	*D*—H⋯*A*
C4—H4⋯S1	0.98	2.65	3.180 (9)	114
C4—H4⋯O3	0.98	2.34	2.895 (10)	115
C2—H2*B*⋯*Cg* ^i^	0.97	2.90	3.807 (8)	156

Symmetry code: (i) 
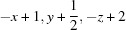
.

**Fig. 1.**
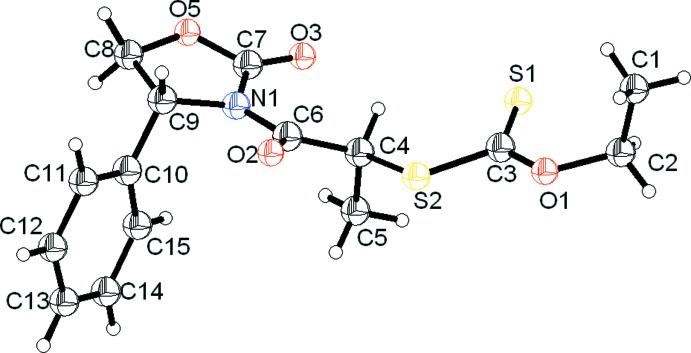
The molecular structure of the title molecule, with atom labelling. Displacement ellipsoids are drawn at the 50% probability level.

**Fig. 2.**
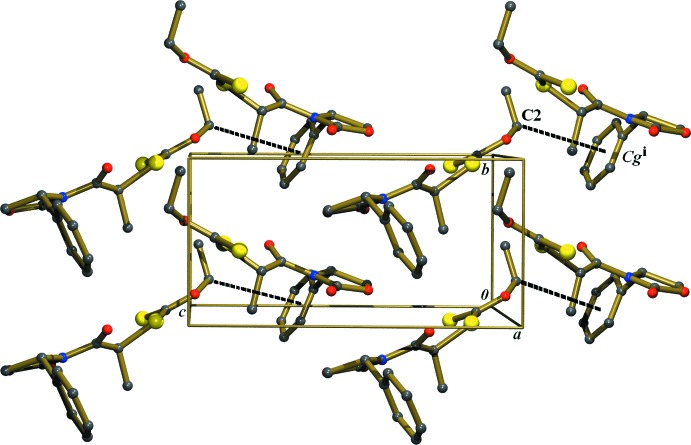
The crystal packing of the title compound, viewed along the a axis. The dashed lines indicate the C—H···π interactions (see Table 1 for details; H atoms not involved in these interactions have been omitted for clarity).

## supplementary crystallographic information

### 1. Comment

Oxazolidinones and their derivatives show interesting chemical and biological activities. The use of chiral oxazolidinones auxiliaries in asymmetric synthesis has found wide application in a variety of stereoselective reactions over the last two decades (Evans, 1982; Ager *et al.*, 1997). In addition oxazolidinonas represent a novel class of synthetic antimicrobial agents, the most promising feature of these compounds is their oral activity against multidrug-resistant Gram-positive bacteria which have created tremendous therapeutic problems in recent years (Müller *et al.*, 1999). Based on this, and as part of our ongoing research program directed toward the synthesis of novel heterocyclic compounds (see for example: López-Ruiz *et al.*, 2011) we report herein on the use of (*R*)-4-phenyloxazolidin-2-one for the synthesis of the title xanthate-oxazilidinone derivative, which has potential applications as a chiral auxiliary in asymmetric reactions.

The title compound was obtained by addition of *O*-Ethylxanthic acid potassium salt to a diastereomeric mixture of (4*R*)-3-(2-Chloropropanoyl)-4-phenyloxazolidin-2-one in acetone.

The absolute configuration of the newly created stereogenic carbon, C4, could be deduced from the relative configuration of carbon atom C9, see Fig. 1. The oxazolidinone ring has a twisted conformation on bond C9—C8 [puckering parameters (Cremer & Pople, 1975), φ = 313 (4)°] similar to the twisted conformation on bond C6—C8 for the axazolidinone ring in the 3-amino-2-oxazolidinone derivatives [φ = 53.6174° and φ = 54.0837°] (Bartczak *et al.*, 2001; Kruszynski *et al.*, 2001) and the same conformation was observed for unsubstituted 2-oxazolidinone (Wouters *et al.*, 1997).

The bond angles around atom N1 in the oxazolidinone ring are in agreement with the observed tendencies for the bond angles in 3,4-disustituted oxazolidinone derivatives (Marsh *et al.*, 1992; Evain *et al.*, 2002; Hwang *et al.*, 2006). The C9—N1, C7—N1 and C6—N1 bond distances, 1.483 (7), 1.382 (8) and 1.411 (8) Å, respectively, are slightly longer than the average values reported for C*sp*^3^—N(3) and C*sp*^2^—N(3) in γ-lactams [C*—N(—C*)—C=O (*endo*) = 1.462 and C*—N(—C*)—C=O = 1.347 Å respectively] (Allen *et al.*, 1987).

The C7═O3 bond length 1.198 (8) Å is slightly shorter than a C*sp*^2^=O(1) in γ-lactams [C*—N(—C*)—C=O = 1.225 Å] and close to normal C*sp*^2^=O(1) in γ-lactones [C*—C(=O)—O—C* = 1.201 Å]. The C8—O4 bond, 1.449 (8) is slightly shorter than the C*sp*^3^—O(2) [C*—O—C(=O) = 1.464 Å] in γ-lactones while C7—O4 bond 1.343 (7) Å is close to normal C*sp*^2^=O(2) in γ-lactones [C*—C(═O)—O—C* = 1.350 Å]. Moreover the C4—S2 and C3=S1 bond distances are 1.811 (6) and 1.642 (7) Å respectively being slightly shorter than C*sp*^3^—S(2) and C*sp*^2^═S(1) [C—CH—S—C = 1.819 and (*X*)_2_—C═S (*X* = C, N, O, S) = 1.671 Å, respectively].

In the crystal, molecules are linked via C-H···π interactions forming zigzag chains along [001]; (Table 1 and Fig. 2).

### 2. Experimental

For the preparation of the title compound, a solution of (4*R*)-4-phenyloxazolidin-2-one (10.70 mmol) in distilled THF (25 ml) was cooled to 195 K under a nitrogen atmosphere, and a solution of n-butyllithium in hexane (12.95 mmol) was added dropwise. After 2-chloropropanoyl chloride (10.79 mmol) was introduced dropwise and stirring was continued at 195 K for 6 h. Then the reaction mixture was diluted with saturated solution of NH_3_SO_4_ and extracted with dichloromethane (3 × 10 ml). The combined organic layers were washed with water and brine, dried over anhydrous Na_2_SO_4_ and concentrated under vacuum. Purification by chromatography column on silica gel (eluent: hexane/ethyl acetate 9:1) gave the diastereomeric mixture of (4*R*)-3- (2-Chloropropanoyl)-4-phenyloxazolidin-2-one in 98% yield. To a solution of this diastereomeric mixture (31.19 mmol) in acetone at 273 K was added the *O*-Ethylxanthic acid potassium salt (46.78 mmol) and the reaction was stirred at room temperature for 12 h. Then the reaction mixture was diluted with a saturated solution of NH_3_SO_4_ and extracted with dichloromethane (3 × 10 ml). The organic layer was dried over anhydrous Na_2_SO_4_ and concentrated under vacuum. Purification by chromatography column on silica gel (eluent: hexane/ethyl acetate 9:1) gave the diastereomeric mixture of (4*R*)-3-((2*R*)-(2-*O*-Ethyl carbonodithioate) propanoyl)-4-phenyloxazolidin-2-one in a 77% of yield. Block-like colourless crystals of the title compound were obtained by slow evaporation of an hexane/ethyl acetate (9:1) solution. Spectroscopic data for the title compound are available in the archived CIF.

### 3. Refinement

The H atoms were inlcuded in calculated positions and treated as riding atoms: C-H = 0.93 - 0.98 Å with U_iso_(H) = 1.5U_eq_(C-methyl) and = 1.2U_eq_(C) for other H atoms.

### Crystal data

**Table:**

C_15_H_17_NO_4_S_2_	*F*(000) = 356
*M**_r_* = 339.42	*D*_x_ = 1.369 Mg m^−^^3^
Monoclinic, *P*2_1_	Mo *K*α radiation, λ = 0.71073 Å
Hall symbol: P 2yb	Cell parameters from 1321 reflections
*a* = 10.8558 (15) Å	θ = 1.7–26.0°
*b* = 6.1867 (9) Å	µ = 0.34 mm^−^^1^
*c* = 12.3057 (17) Å	*T* = 293 K
β = 94.911 (4)°	Block, colourless
*V* = 823.4 (2) Å^3^	0.2 × 0.17 × 0.16 mm
*Z* = 2	

### Data collection

**Table:**

Bruker SMART CCD diffractometer	1681 reflections with *I* > 2σ(*I*)
Radiation source: fine-focus sealed tube	*R*_int_ = 0.152
Graphite monochromator	θ_max_ = 26.0°, θ_min_ = 1.7°
φ and ω scans	*h* = −13→13
10191 measured reflections	*k* = −7→7
3223 independent reflections	*l* = −15→15

### Refinement

**Table:**

Refinement on *F*^2^	Secondary atom site location: difference Fourier map
Least-squares matrix: full	Hydrogen site location: inferred from neighbouring sites
*R*[*F*^2^ > 2σ(*F*^2^)] = 0.067	H-atom parameters constrained
*wR*(*F*^2^) = 0.191	*w* = 1/[σ^2^(*F*_o_^2^) + (0.0945*P*)^2^] where *P* = (*F*_o_^2^ + 2*F*_c_^2^)/3
*S* = 0.87	(Δ/σ)_max_ < 0.001
3223 reflections	Δρ_max_ = 0.38 e Å^−^^3^
201 parameters	Δρ_min_ = −0.35 e Å^−^^3^
1 restraint	Absolute structure: Flack (1983), 6968 Friedel pairs
Primary atom site location: structure-invariant direct methods	Absolute structure parameter: 0.08 (18)

### Special details

**Table:**

Experimental. Spectroscopic data for the title compound: ^1^H NMR (400 MHz, CDCl_3_)δ: 7.2 (m, 5H, CH, arom), 5.6 (q, 1H, CH (H-4), *J*= 7.3 Hz), 5.4 (dd, CH (H-9), *J^1^*= 3.2 Hz, *J^2^*=8.6 Hz), 4.7 (t, 1H (H-8a), CH_2_, *J*= 8.8 Hz), 4.6 (m, 2H, CH_2_(H-2)), 4.3 (dd, 1H (H-8 b), *J^1^*= 3.2 Hz, *J^2^*= 8.6 Hz), 1.4 (d,3*H*,CH_3_ (H-5), *J*= 7.3 Hz), 1.4 (t, 3H,CH_3_ (H-1), *J*= 7.3 Hz); ^13^C NMR (100 MHz, CDCl_3_) δ: 213 (CS), 171.05 (CO), 153.53 (CO), 138.74 (C), 129.33 (CH), 128.93 (2CH), 125.90 (CH), 70.35 (2CH_2_), 58.03 (CH), 57.94 (CH), 47.20 (CH), 15.80 (CH_3_), 13.60 (CH_3_).
Geometry. All s.u.'s (except the s.u. in the dihedral angle between two l.s. planes) are estimated using the full covariance matrix. The cell s.u.'s are taken into account individually in the estimation of s.u.'s in distances, angles and torsion angles; correlations between s.u.'s in cell parameters are only used when they are defined by crystal symmetry. An approximate (isotropic) treatment of cell s.u.'s is used for estimating s.u.'s involving l.s. planes.
Refinement. Refinement of *F*^2^ against ALL reflections. The weighted *R*-factor *wR* and goodness of fit *S* are based on *F*^2^, conventional *R*-factors *R* are based on *F*, with *F* set to zero for negative *F*^2^. The threshold expression of *F*^2^ > 2σ(*F*^2^) is used only for calculating *R*-factors(gt) *etc*. and is not relevant to the choice of reflections for refinement. *R*-factors based on *F*^2^ are statistically about twice as large as those based on *F*, and *R*- factors based on ALL data will be even larger.

### Fractional atomic coordinates and isotropic or equivalent isotropic displacement parameters (Å^2^)

**Table:**

	*x*	*y*	*z*	*U*_iso_*/*U*_eq_	
C1	0.8431 (7)	0.9153 (19)	1.0271 (7)	0.101 (3)	
H1A	0.8415	0.9226	0.9491	0.151*	
H1B	0.9209	0.9693	1.0591	0.151*	
H1C	0.7772	1.0015	1.0513	0.151*	
C2	0.8275 (6)	0.6889 (16)	1.0609 (6)	0.082 (2)	
H2A	0.8932	0.6011	1.0354	0.098*	
H2B	0.8328	0.6804	1.1399	0.098*	
C3	0.6967 (6)	0.5034 (10)	0.9216 (5)	0.0560 (17)	
C4	0.5331 (5)	0.2814 (10)	0.7734 (5)	0.0479 (15)	
H4	0.6011	0.3210	0.7298	0.057*	
C5	0.5440 (7)	0.0432 (10)	0.8030 (6)	0.069 (2)	
H5A	0.4763	0.0023	0.8439	0.103*	
H5B	0.6206	0.0185	0.8462	0.103*	
H5C	0.5421	−0.0415	0.7375	0.103*	
C6	0.4117 (6)	0.3295 (9)	0.7086 (5)	0.0459 (14)	
C7	0.4827 (6)	0.1846 (9)	0.5343 (5)	0.0481 (15)	
C8	0.2958 (6)	0.1740 (13)	0.4298 (5)	0.0613 (17)	
H8A	0.2566	0.0348	0.4153	0.074*	
H8B	0.2653	0.2748	0.3734	0.074*	
C9	0.2687 (5)	0.2584 (10)	0.5439 (4)	0.0458 (14)	
H9	0.2434	0.4104	0.5388	0.055*	
C10	0.1727 (5)	0.1287 (9)	0.5970 (4)	0.0420 (14)	
C11	0.0555 (5)	0.2134 (10)	0.6019 (5)	0.0516 (16)	
H11	0.0357	0.3479	0.5715	0.062*	
C12	−0.0328 (6)	0.0959 (11)	0.6529 (6)	0.0632 (19)	
H12	−0.1116	0.1528	0.6567	0.076*	
C13	−0.0047 (6)	−0.1024 (13)	0.6975 (5)	0.066 (2)	
H13	−0.0641	−0.1776	0.7324	0.080*	
C14	0.1105 (7)	−0.1919 (11)	0.6912 (6)	0.0673 (19)	
H14	0.1292	−0.3279	0.7204	0.081*	
C15	0.1990 (5)	−0.0740 (13)	0.6399 (5)	0.0583 (15)	
H15	0.2770	−0.1332	0.6346	0.070*	
N1	0.3934 (4)	0.2412 (7)	0.6027 (4)	0.0423 (11)	
O1	0.7069 (4)	0.6056 (9)	1.0155 (4)	0.0770 (15)	
O2	0.3286 (4)	0.4268 (9)	0.7448 (3)	0.0631 (11)	
O3	0.5918 (4)	0.1651 (7)	0.5559 (4)	0.0566 (12)	
O4	0.4291 (4)	0.1544 (7)	0.4330 (3)	0.0551 (11)	
S1	0.80766 (14)	0.4339 (4)	0.84599 (14)	0.0760 (6)	
S2	0.53803 (14)	0.4534 (3)	0.89279 (13)	0.0631 (5)	

### Atomic displacement parameters (Å^2^)

**Table:**

	*U*^11^	*U*^22^	*U*^33^	*U*^12^	*U*^13^	*U*^23^
C1	0.068 (5)	0.143 (8)	0.091 (6)	−0.024 (6)	−0.002 (4)	0.019 (7)
C2	0.054 (4)	0.126 (8)	0.063 (4)	0.001 (4)	−0.010 (4)	−0.019 (5)
C3	0.049 (3)	0.072 (5)	0.046 (4)	0.001 (3)	0.002 (3)	0.003 (3)
C4	0.040 (3)	0.054 (4)	0.051 (3)	0.004 (3)	0.011 (3)	−0.003 (3)
C5	0.081 (5)	0.067 (5)	0.057 (4)	0.006 (4)	0.000 (4)	0.006 (3)
C6	0.048 (3)	0.043 (3)	0.048 (4)	−0.006 (3)	0.011 (3)	−0.002 (3)
C7	0.057 (4)	0.038 (3)	0.051 (4)	−0.003 (3)	0.016 (3)	0.002 (3)
C8	0.053 (4)	0.083 (5)	0.048 (4)	−0.002 (3)	0.005 (3)	−0.002 (4)
C9	0.041 (3)	0.050 (3)	0.046 (3)	−0.002 (3)	0.005 (3)	0.002 (3)
C10	0.045 (3)	0.039 (3)	0.042 (3)	−0.004 (3)	0.003 (3)	−0.003 (3)
C11	0.049 (4)	0.056 (4)	0.049 (4)	0.007 (3)	0.003 (3)	−0.002 (3)
C12	0.040 (3)	0.076 (5)	0.075 (5)	−0.007 (4)	0.010 (3)	−0.013 (4)
C13	0.056 (4)	0.085 (6)	0.060 (4)	−0.034 (4)	0.016 (3)	−0.003 (4)
C14	0.060 (4)	0.070 (5)	0.071 (4)	−0.009 (4)	0.000 (4)	0.007 (4)
C15	0.042 (3)	0.060 (4)	0.073 (4)	−0.001 (4)	0.010 (3)	−0.003 (4)
N1	0.034 (2)	0.050 (3)	0.044 (3)	0.000 (2)	0.009 (2)	0.000 (2)
O1	0.049 (3)	0.116 (4)	0.065 (3)	−0.009 (3)	0.003 (2)	−0.028 (3)
O2	0.047 (2)	0.088 (3)	0.055 (2)	0.014 (3)	0.0065 (19)	−0.019 (3)
O3	0.044 (3)	0.065 (3)	0.063 (3)	0.006 (2)	0.015 (2)	−0.006 (2)
O4	0.059 (3)	0.061 (3)	0.047 (3)	−0.001 (2)	0.017 (2)	−0.004 (2)
S1	0.0462 (9)	0.1207 (15)	0.0621 (10)	−0.0011 (13)	0.0095 (7)	−0.0115 (13)
S2	0.0438 (8)	0.0872 (12)	0.0585 (9)	−0.0036 (10)	0.0067 (7)	−0.0216 (10)

### Geometric parameters (Å, º)

**Table:**

C1—C2	1.475 (13)	C7—O4	1.344 (7)
C1—H1A	0.9600	C7—N1	1.382 (7)
C1—H1B	0.9600	C8—O4	1.449 (8)
C1—H1C	0.9600	C8—C9	1.550 (8)
C2—O1	1.472 (8)	C8—H8A	0.9700
C2—H2A	0.9700	C8—H8B	0.9700
C2—H2B	0.9700	C9—N1	1.483 (7)
C3—O1	1.313 (7)	C9—C10	1.507 (8)
C3—S1	1.642 (6)	C9—H9	0.9800
C3—S2	1.756 (6)	C10—C15	1.381 (9)
C4—C6	1.511 (8)	C10—C11	1.383 (8)
C4—C5	1.520 (8)	C11—C12	1.395 (9)
C4—S2	1.811 (6)	C11—H11	0.9300
C4—H4	0.9800	C12—C13	1.367 (10)
C5—H5A	0.9600	C12—H12	0.9300
C5—H5B	0.9600	C13—C14	1.376 (10)
C5—H5C	0.9600	C13—H13	0.9300
C6—O2	1.201 (7)	C14—C15	1.399 (9)
C6—N1	1.411 (7)	C14—H14	0.9300
C7—O3	1.198 (8)	C15—H15	0.9300
			
C2—C1—H1A	109.5	C9—C8—H8A	110.6
C2—C1—H1B	109.5	O4—C8—H8B	110.6
H1A—C1—H1B	109.5	C9—C8—H8B	110.6
C2—C1—H1C	109.5	H8A—C8—H8B	108.7
H1A—C1—H1C	109.5	N1—C9—C10	112.7 (5)
H1B—C1—H1C	109.5	N1—C9—C8	100.5 (4)
O1—C2—C1	110.2 (6)	C10—C9—C8	113.9 (5)
O1—C2—H2A	109.6	N1—C9—H9	109.8
C1—C2—H2A	109.6	C10—C9—H9	109.8
O1—C2—H2B	109.6	C8—C9—H9	109.8
C1—C2—H2B	109.6	C15—C10—C11	119.2 (5)
H2A—C2—H2B	108.1	C15—C10—C9	121.4 (5)
O1—C3—S1	127.9 (5)	C11—C10—C9	119.3 (5)
O1—C3—S2	105.7 (4)	C10—C11—C12	119.5 (6)
S1—C3—S2	126.4 (4)	C10—C11—H11	120.2
C6—C4—C5	111.4 (5)	C12—C11—H11	120.2
C6—C4—S2	106.1 (4)	C13—C12—C11	120.6 (6)
C5—C4—S2	112.3 (5)	C13—C12—H12	119.7
C6—C4—H4	109.0	C11—C12—H12	119.7
C5—C4—H4	109.0	C12—C13—C14	120.8 (6)
S2—C4—H4	109.0	C12—C13—H13	119.6
C4—C5—H5A	109.5	C14—C13—H13	119.6
C4—C5—H5B	109.5	C13—C14—C15	118.5 (7)
H5A—C5—H5B	109.5	C13—C14—H14	120.7
C4—C5—H5C	109.5	C15—C14—H14	120.7
H5A—C5—H5C	109.5	C10—C15—C14	121.3 (6)
H5B—C5—H5C	109.5	C10—C15—H15	119.4
O2—C6—N1	119.1 (5)	C14—C15—H15	119.4
O2—C6—C4	123.5 (5)	C7—N1—C6	127.6 (5)
N1—C6—C4	117.3 (5)	C7—N1—C9	112.3 (5)
O3—C7—O4	122.3 (5)	C6—N1—C9	118.2 (4)
O3—C7—N1	128.5 (6)	C3—O1—C2	120.5 (5)
O4—C7—N1	109.3 (5)	C7—O4—C8	111.5 (5)
O4—C8—C9	105.7 (5)	C3—S2—C4	103.2 (3)
O4—C8—H8A	110.6		
			
C5—C4—C6—O2	108.2 (7)	O3—C7—N1—C9	177.2 (6)
S2—C4—C6—O2	−14.3 (7)	O4—C7—N1—C9	−1.9 (6)
C5—C4—C6—N1	−67.3 (7)	O2—C6—N1—C7	158.5 (6)
S2—C4—C6—N1	170.3 (4)	C4—C6—N1—C7	−25.8 (8)
O4—C8—C9—N1	−8.7 (6)	O2—C6—N1—C9	−4.6 (8)
O4—C8—C9—C10	−129.5 (5)	C4—C6—N1—C9	171.1 (5)
N1—C9—C10—C15	−40.0 (8)	C10—C9—N1—C7	128.3 (5)
C8—C9—C10—C15	73.6 (7)	C8—C9—N1—C7	6.7 (6)
N1—C9—C10—C11	140.7 (5)	C10—C9—N1—C6	−66.1 (6)
C8—C9—C10—C11	−105.7 (6)	C8—C9—N1—C6	172.3 (5)
C15—C10—C11—C12	1.9 (9)	S1—C3—O1—C2	5.3 (10)
C9—C10—C11—C12	−178.7 (5)	S2—C3—O1—C2	−175.1 (6)
C10—C11—C12—C13	−0.3 (10)	C1—C2—O1—C3	92.6 (8)
C11—C12—C13—C14	−1.2 (10)	O3—C7—O4—C8	176.3 (6)
C12—C13—C14—C15	1.1 (10)	N1—C7—O4—C8	−4.5 (6)
C11—C10—C15—C14	−2.0 (9)	C9—C8—O4—C7	8.6 (7)
C9—C10—C15—C14	178.6 (6)	O1—C3—S2—C4	−172.4 (5)
C13—C14—C15—C10	0.5 (10)	S1—C3—S2—C4	7.2 (5)
O3—C7—N1—C6	13.3 (10)	C6—C4—S2—C3	−149.4 (4)
O4—C7—N1—C6	−165.8 (5)	C5—C4—S2—C3	88.8 (5)

### Hydrogen-bond geometry (Å, º)

Cg is the centroid of the C10–C15 phenyl ring.

**Table:**

*D*—H···*A*	*D*—H	H···*A*	*D*···*A*	*D*—H···*A*
C4—H4···S1	0.98	2.65	3.180 (9)	114
C4—H4···O3	0.98	2.34	2.895 (10)	115
C2—H2*B*···*Cg*^i^	0.97	2.90	3.807 (8)	156

Symmetry code: (i) −*x*+1, *y*+1/2, −*z*+2.
